# “It’s more than just the act of not using. It’s a feeling of finally completing something.”: Person-centered definitions of successful treatment outcomes from patients and staff at a methadone treatment program in Baltimore City

**DOI:** 10.1016/j.josat.2025.209683

**Published:** 2025-03-20

**Authors:** Valerie D. Bradley, Mary B. Kleinman, Morgan S. Anvari, Tolulope M. Abidogun, C.J. Seitz-Brown, Annabelle M. Belcher, Thomas O. Cole, Aaron D. Greenblatt, Jessica F. Magidson

**Affiliations:** a Department of Psychology, University of Maryland-College Park, College Park, MD, USA; b Department of Psychiatry, University of Maryland School of Medicine, Baltimore, MD, USA; c Center for Substance Use, Addiction & Health Research (CESAR), University of Maryland, College Park, College Park, MD, USA; d College of Public Health, George Mason University, Fairfax, VA, USA

**Keywords:** Opioid use disorder, Person-centered, Methadone treatment, Treatment outcomes, Treatment success

## Abstract

**Background:**

Successful outcomes in substance use disorder (SUD) treatment are traditionally defined by retention in care, substance use cessation, and relapse prevention. However, these metrics may overlook important, person-centered aspects of success, especially for marginalized populations facing significant inequities in care. With overdose-related deaths disproportionately affecting racially minoritized groups, understanding a more inclusive definition of success in opioid use disorder (OUD) treatment is essential for improving clinical treatment, research, and policy. This study explores how patients and staff define successful treatment outcomes and how those definitions align or differ at an outpatient methadone treatment program in Baltimore City.

**Methods:**

We conducted qualitative interviews and focus groups with 32 participants, including patients, clinical staff, and peer recovery specialists (PRSs) at a methadone treatment program serving primarily low-income, racially minoritized individuals with OUD. The sample was 59 % male, average age 48.7, and 66 % Black or African American. Semi-structured interview guides prompted patients (*n* = 20) and clinical staff/PRSs (*n* = 12) to describe successful treatment experiences. Data were analyzed using thematic analysis and contextualized within the Health Equity Implementation Framework to propose influences on equitable methadone treatment that optimize treatment outcomes.

**Results:**

Five key themes emerged in defining person-centered successful treatment outcomes: (1) improvements in general health, (2) productivity and accomplishment, (3) social improvements, (4) substance use changes, and (5) treatment engagement. Patients and staff agreed on two-thirds of subthemes but showed notable differences. Patients emphasized experiential and social aspects of recovery, such as increased activities, supporting others, and resisting substance use influences. Staff focused on sustained behavioral change and long-term recovery milestones, including substance use behaviors, self-worth, community involvement, and treatment planning.

**Conclusion:**

Person-centered definitions of success in methadone treatment encompass a range of psychosocial, behavioral, and health-related factors. While patients and staff agreed on overall themes, their perspectives diverged on several subthemes. Incorporating diverse patient perspectives in defining methadone treatment success, particularly from marginalized groups, is essential for improving research, policy, and clinical practices to enhance patient experiences and outcomes in methadone treatment.

## 1. Introduction

In the face of an increasingly lethal opioid overdose crisis, with largely opioid-driven overdose deaths surpassing 112,000 in the 12 months preceding May 2023 (Ahmad et al., 2024), medications for opioid use disorder (MOUD) such as methadone, buprenorphine, and naltrexone treatments offer a highly effective means of treatment for individuals with opioid use disorder (Sofuoglu et al., 2019). Despite proven efficacy, maintaining patient engagement in care remains challenging (Morgan et al., 2018). Nationally, retention rates at six months post-intake stand below 50 % (A. Williams et al., 2017; A. R. Williams et al., 2019), with even lower retention rates among low-income, racially minoritized individuals (Manhapra et al., 2017; Samples et al., 2018; Stahler & Mennis, 2018; Weinstein et al., 2017).

Early treatment discontinuation presents a significant obstacle to the effectiveness of treatment approaches that involve MOUD and correlate with increased mortality rates (Degenhardt et al., 2009; Stafford et al., 2022). Research has pointed to discord between opioid treatment program (OTP) policies and patients’ perception of treatment gains, where patients observe successes in life areas peripheral to the traditional program goals. Clinical encounters in MOUD programs, particularly in highly structured treatment modalities such as methadone treatment, have been shown to play a critical role in patient engagement in care. Studies have shown that a lack of individualized treatment approaches and disempowering patient-provider interactions drive patient discontent, and treatment dissatisfaction has been linked to increases in treatment departure and discharge (Austin et al., 2024; Kelly et al., 2011). This discordance can divide patients and staff, contributing to treatment discontent, patient discontinuation, early treatment discharge, and the demoralization of program staff experiencing case-load turnovers, frustration, and a sense of professional setback (Mitchell et al., 2011). To address this disconnect, this study presents the voices of historically marginalized individuals with opioid use disorder and treatment staff as they describe first-hand experiences and observations of patient-defined treatment success at a methadone treatment program in the pursuit of a more person-centered definition of success.

Understanding and incorporating patients’ perspectives may be vital to optimizing patient experiences and improving outcomes in treatment approaches that include MOUD (Cernasev et al., 2021). Traditional metrics of substance use treatment and MOUD success identified by staff, researchers, and policymakers—namely substance use abstinence, treatment retention, and the prevention of recurrent substance use often described as relapse—fall short of capturing the full range of treatment successes as experienced by patients themselves (Biondi et al., 2020; Fishman et al., 2020; A. R. Williams et al., 2018, Williams et al., 2019). Substance use and treatment adherence challenges can often serve as the rationale for the administrative discharge of a patient (Mitchell et al., 2011). Further, patients who find treatment programs inflexible to their needs and unreceptive to their goals may initiate treatment discharge (Mitchell et al., 2011). As a result, researchers have begun to explore patients’ goals in MOUD treatment (Cioe et al., 2020; Hooker et al., 2022; Sanger et al., 2022).

Research examining addiction treatment inclusive of MOUD has not yet codified a person-centered conceptualization of treatment success. Patient perspectives—particularly the perspectives of low-income, minoritized populations engaged in methadone treatment—remain largely unexplored. The stringent focus on substance use abstinence in treatment overlooks the importance of addressing patient-specific goals in treatment (Lopez et al., 2022). Shifting the focus from abstinence and treatment attendance to patient-defined indicators of success enables staff to effectively align treatment planning with patient values and goals (Mitchell et al., 2011). Addiction treatment programs that provide MOUD may see greater engagement and retention when patient goals are represented in treatment plans (Joseph et al., 2021), and researchers may be able to better measure the effectiveness of treatment by assessing the full range of treatment outcomes (Sanger et al., 2022).

While numerous evidence-based practices exist for treating substance use disorders like OUD, including cognitive behavioral therapy, relapse prevention strategies, motivational interviewing, contingency management, couples/family counseling, and group therapy, medication for opioid use disorder MOUD has proven to be a life-saving, frontline treatment for opioid use disorder. Engagement in MOUD treatment often includes opportunities for adjacent care such as behavioral treatment, addiction counseling, and other services (Glasner-Edwards & Rawson, 2010; Madras, Ahmad, Wen, & Sharfstein, 2020). The Food and Drug Administration currently approves three medications for patients seeking opioid use treatment with a medication component. Methadone, buprenorphine, and naltrexone reduce opioid withdrawal symptoms and cravings and block the euphoric effects of opioids. While buprenorphine and naltrexone are often prescribed in primary care settings, methadone treatment is a more stringently supervised form of MOUD administered in opioid treatment program (OTP) settings only (Committee on Medication-Assisted Treatment for Opioid Use Disorder et al., 2019).

As part of methadone treatment, OTPs are federally mandated to provide services such as behavioral counseling and case management, as well as options for services such as medical, educational, vocational, and other resources (Madras, Ahmad, Wen, & Sharfstein, 2020; Substance Abuse and Mental Health Services Administration (SAMHSA), 2024). The medication component of methadone treatment consists of daily supervised dosing at an outpatient treatment program, where patients engage in regular counseling sessions and receive limited take-home bottles allocated based on narrow benchmarks of success. Evidence suggests that patients are at significant risk for adverse events and elevated mortality following discharge from methadone treatment, and barriers associated with methadone treatment have been linked to treatment discontinuation (Berk et al., 2025; Pasman et al., 2022; Thakrar et al., 2023). While patients who remain in methadone treatment for longer periods have shown similar mortality rates compared to other forms of MOUD, high rates of early treatment discontinuation in methadone treatment remain a significant barrier to treatment effectiveness (Cousins et al., 2016; Gottlieb et al., 2023).

The need to improve person-centered methadone treatment has been recognized at the federal level; in January of 2024, the Substance Abuse and Mental Health Services Administration (SAMHSA) made permanent the previously temporary reduction in methadone treatment restrictions during the COVID–19 Public Health Emergency. These newly codified measures reduce barriers to take-home methadone, emphasize collaborative patient-staff decision-making, and remove the restrictive time-frames dictating methadone treatment eligibility, such as length of opioid addiction history and number of prior treatment attempts (Substance Abuse and Mental Health Services Administration (SAMHSA), 2024). Per SAMSHA guidelines, licensed OTP medical practitioners assess several factors to determine take-home bottle eligibility. These criteria include the absence of active substance use disorders which may increase the patient’s risk of overdose, regular attendance for supervised medication administration, appropriate conduct within the clinic, responsible use and storage of methadone medication, and any other considerations deemed relevant by the medical director (Substance Abuse and Mental Health Services Administration (SAMHSA), 2024). Incorporating patient goals in treatment and establishing more person-centered metrics of methadone treatment success is paramount to achieving SAMHSA’s goal of providing person-centered care and can aid in interpreting patient success in treatment beyond the assessment of take-home bottle eligibility.

While redefining success in MOUD based on patient-defined goals may improve patient outcomes, few studies have explored patient experiences of success in methadone treatment specifically (Goedel et al., 2020; Hansen et al., 2013; Madras, Ahmad, Wen, & Sharfstein, 2020). Further, to our knowledge, no studies have sought the perspectives of low-income, racially minoritized individuals engaged in methadone treatment. Methadone treatment must include the perspective of marginalized patients who face disproportionate harms in the opioid crisis, are the most likely to experience systemic inequalities in healthcare, and whose voices have been historically excluded from research in methadone treatment administration (Burlew et al., 2011; Hollander et al., 2021; Hulsey, 2022; Jordan et al., 2021; Lagisetty et al., 2019; National Academies of Sciences et al., 2019; Priest et al., 2022).

Incorporating a multifaceted understanding of methadone treatment success from the perspectives of minoritized groups may have important implications for developing more equitable, person-centered policies and treatment programs. As such, this study aimed to understand key stakeholders’ perspectives on successful methadone treatment outcomes at a community-based methadone treatment program serving predominantly low-income, racially minoritized patients in Baltimore. Specifically, we sought to understand 1) patients’ descriptions of successful methadone treatment outcomes, 2) staff observations of how patients experience success in methadone treatment, and 3) overlap and divergence in how each group defines methadone treatment success.

## 2. Methods

### 2.1. Setting

This study was conducted at the University of Maryland Addiction Treatment Center, a large, community-based outpatient OTP in West Baltimore with an average daily census of over five hundred patients. Study activities took place from September through December 2019. The peripheral services offered alongside methadone medication included primary care, infectious disease care, psychiatry, psychological counseling, addiction counseling, individual and group therapy, home health services, social worker services, peer recovery specialist services, and case management. The Baltimore-based OTP abided by SAMHSA regulations and guidelines for methadone treatment programmatic structure (Substance Abuse and Mental Health Services Administration (SAMHSA), 2024). At the time of this study, methadone treatment was dispensed daily (Monday through Saturday) with a take-home dose provided on Sunday. Per SAMHSA regulations, admitted patients were required to complete monthly drug testing and follow program rules. Patients who missed 30 or more consecutive days of attendance were administratively discharged. Following the pre-COVID SAMHSA guidelines, staff prescribed take-home doses dependent on treatment adherence (i.e., 90 consecutive days of negative toxicology screens for alcohol and drug use).

### 2.2. Participants and recruitment

Participants recruited for this study (*N* = 32) included patients currently enrolled in the methadone treatment program (*n* = 20) and two levels of staff: OTP-based clinical staff (*n* = 8) and peer recovery specialists (PRSs) working in this and other recovery programs in Baltimore City (*n* = 4). Participants primarily identified as male (59 %) and Black or African American (66 %) [see Table 1 for sociodemographic characteristics of participants].

The study recruited participants using flyers, announcements, word of mouth, and voluntary referrals. Clinical staff purposefully identified and referred patients to the study based on either demonstrated challenges with methadone treatment adherence or consistent methadone treatment adherence in order to capture a range of patient perspectives and experiences. Clinical staff participants included drug treatment counselors (*n* = 6), a social worker (*n* = 1), a nurse practitioner (*n* = 1), and a PRS (*n* = 1). Community-based PRSs (*n* = 3) in Baltimore City learned about the study through announcements and outreach conducted by a trained peer research collaborator at PRS agencies and other community-based organizations. The study recruited PRSs based on their work in treatment and community settings across Baltimore City, and all PRSs were familiar with and had experience working with populations of patients receiving medications for OUD treatment. During recruitment and informed consent, all program staff and patient participants were informed that study participation was optional, confidential, and would not affect their treatment or employment at the OTP.

Trained members of the research team administered informed consent to all participants at the time of enrollment. Participants were provided the option to read the consent form or to have it read to them. The University of Maryland-College Park Institutional Review Board verified that all consent forms and study documents were written at a reading level and language complexity appropriate for the targeted participant population. During informed consent, participants were informed of all potential study risks and benefits and reminded that they could leave the study at any point. Before completing informed consent, researchers were trained to ask three structured questions about the consent form as part of the evaluation to sign the consent, ensuring that the participants understood the nature of the research based on the consent form and had all of their questions answered.

The study offered participants the option to participate in either focus groups or individual interviews that aimed to capture the patient and staff perspectives as fully as possible and, recognizing that the two qualitative methodologies may elicit different responses, to accommodate varying work schedules and personal preferences. Three staff focus groups and two patient focus groups took place with a maximum of six participants in each. Participants chose to participate in a focus group (*n* = 22) or individual interview (*n* = 10) but could not engage in both. Focus groups were separated such that patients only participated with other patients and staff only participated with other staff. Participants received a $25 gift card compensation for their participation in the interview or focus group. As recommended in qualitative research, the number of individuals needed to reach theoretical saturation informed the sample size (Pope & Mays, 2006). After recruiting 32 participants, redundancy emerged and no new themes central to the study question arose, indicating theoretical saturation.

### 2.3. Procedures

The research team developed semi-structured guides for focus groups and individual interviews that were informed by prior literature on substance use recovery (Ashford et al., 2019). The interview and focus group facilitator team included a doctoral-level clinical supervisor, a graduate research assistant, and a post-baccalaureate research assistant. The principal investigator trained all facilitators in qualitative interviewing, including specific training on administering the semi-structured interview and focus group guides. One of the three facilitators conducted each interview, and two facilitators co-led focus groups. Facilitators had no prior involvement at the addiction treatment center and were not familiar with any participants at the time of enrollment.

The interview/focus group guide questions were intentionally broad and aimed to capture participants’ conceptualization of success in methadone treatment. For example, interview/focus group guides prompted patients to describe their personal perspectives on success in methadone treatment, including describing a time when they were successful in treatment or describing what success would look and feel like. Staff were asked to describe patients they have worked with who have discussed doing well in methadone treatment and discuss what success looked like to those individuals. When participants expressed uncertainty, asked for clarification, or provided brief or unclear responses, interviewers or focus group leaders provided prompts based on prior literature suggesting three core elements of treatment success: sobriety, personal health, and seeking the betterment of one’s community (Ashford et al., 2019; Inanlou et al., 2020) while emphasizing that the focus was on the individual’s perception of treatment success. All individual interviews and focus groups were audio recorded with participant permission and the recordings were later transcribed and reviewed for accuracy. Study procedures were reviewed and approved by the University of Maryland, College Park Institutional Review Board (IRB), which also approved an Interagency Agreement (IAA) with the University of Maryland, Baltimore.

### 2.4. Data analysis

#### 2.4.1. Transcription and coding

A team of trained coders analyzed transcripts using codebook/template thematic analysis (Brooks et al., 2015), a deductive-inductive approach. The coding team consisted of four researchers, including two undergraduate research assistants, one post-baccalaureate research assistant, and a graduate research assistant as trainer and arbiter. The graduate research assistant received training from the study PI and provided training to all other coders. All coders participated in developing a preliminary codebook by deductively coding themes from three transcripts based on the semi-structured interview guide questions that asked about experiences and observations of successful methadone treatment outcomes. The coding team then iteratively developed two codebooks outlining themes, subthemes, and definitions identified in the transcripts based on participant responses. The team modified codebooks (one for patient and one for staff responses) as new concepts arose (Boyatzis, 1998).

Teams of two researchers coded each transcript independently using NVivo Version 12, such that not all researchers reviewed every transcript. For each transcript, two researchers coded independently and met weekly to discuss and resolve discrepancies in coding. Through discussion, coders arrived at a consensus on each code determination. A third-person arbiter engaged in these weekly meetings, and the resolution of coding discrepancies brought to these meetings occurred through discussion and consensus. Through this process, the team coded all final transcripts to complete consensus.

#### 2.4.2. Health equity implementation framework

The Health Equity Implementation Framework (HEIF) guided data analysis and the interpretation of thematic findings. The HEIF explores determinants of equitable healthcare, such as access to, quality of, and outcomes of healthcare innovations, as well as the broader contexts influencing health equity (Woodward et al., 2019). The HEIF considers factors affecting health equity implementation at multiple levels, including the innovation level (e.g., methadone treatment), recipient level (e.g., patients and staff), and the clinical encounter level (e.g., the patient-staff interaction). The HEIF assesses influences on health equity implementation across multiple contexts, such as the inner local context (e.g., a methadone treatment clinic), inner organizational context (e.g., a hospital system), outer healthcare context (e.g., the broader healthcare system), and the societal context (e.g., physical infrastructure, economy, sociopolitical forces; Woodward et al., 2019, 2021).

We contextualized our findings within the HEIF by examining the person-centered definitions of methadone treatment success emerging from this study within the various contexts influencing equity in methadone treatment success. Our utilization of the HEIF was informed by prior literature examining and utilizing the HEIF in which researchers explored various health interventions in the context of HEIF-defined domains to inform equitable implementation in healthcare (Woodward et al., 2021, Brownson et al., 2021; McLoughlin & Martinez, 2022). For example, Drake et al. (2021) applied the HEIF framework to identify barriers and inform more equitable social needs screening in healthcare, offering valuable insights to enhancing treatment delivery (Drake et al., 2021). We applied this framework methodology to methadone treatment settings by examining the results of this study within the relevant individual, clinical, and societal domains and developed recommendations to improve equitable implementation of methadone treatment incorporating a person-centered approach.

## 3. Results

Participants (patients and staff) discussed numerous themes indicative of successful methadone treatment in this study. In line with our aims, we present two primary perspectives of successful treatment outcomes, including patients’ definitions of successful treatment outcomes and staff observations of patients’ experiences of success in methadone treatment. Based on interview and focus group feedback, patient and staff participants agreed on five primary themes to define successful treatment outcomes: (1) improved health and wellbeing; (2) productivity and accomplishment; (3) improved social relationships; (4) changes in substance use-related behaviors; and (5) increased treatment engagement.

### 3.1. Theme 1: health improvements

Patient and staff participants endorsed general health improvements as part of success in methadone treatment, including improved mental and physical well-being and the ability to address health problems. Both groups were aligned across these three health-related subthemes with no significant distinctions.

#### 3.1.1. Patient perspectives

Patient participants described improvements in mental and physical health as critical metrics of success in methadone treatment. Patients explained how succeeding in treatment consisted of being of “sound mind and body,” “experiencing clarity,” and being “as healthy and sustainable as possible.” Patients discussed increased motivation, positivity, focus, and an overall improvement in day-to-day affect as success. When asked what doing well in a treatment program looked like, one patient detailed how they “start[ed] to feel better about [themself] and look better*.”* Participants also described feeling “motivated” and having “that energy where you weren’t exhausted or mentally exhausted.”

A renewed awareness of one’s health challenges and focus on self-care also arose as indicators of success in treatment in the patient group. Patients described how the effects of substance use could not only bring about, but also mask, mental and physical ailments. To some, success in methadone treatment meant a renewed ability to identify and address health-related issues, resulting in improved eating habits, weight, energy levels, focus, and medical attention when required. One participant shared, “I wasn’t eating right at all, and now it’s easier to focus on doing more of the right things … rather than worrying about my next fix” (Interview, Patient, White, Male). Another patient stated, “Now I’m getting done what I really need to get done as far as my health” (Interview, Patient, Black/African American, Female). Most patients agreed that mental and physical health improvements, increased self-care, and health awareness were core to their definition of treatment success.

#### 3.1.2. Staff perspectives

Staff also observed patients citing mental and physical health improvements as part of methadone treatment success. Specifically, staff described physical health improvements such as healthier eating, purposeful exercise, and weight gain. Further, like patients, staff discussed patients’ renewed ability to recognize and attend to health conditions that were no longer masked by the effects of substance use.

A person will enter into an active state of recovery, […] they’re going to have immediate concerns almost right away. […] People will start becoming more concerned with oral health when it comes time to start applying for jobs and re-engaging with intimate relationships.

(FG, Peer, White, Male).

In addition to immediate health needs, staff observed patients experiencing longer-term health benefits, such as regular exercise and engagement in comprehensive healthcare beyond the scope of the methadone treatment program. As indicated by a PRS participant, “that’s when those values really start coming back to the person…they start caring about themselves” (FG, Peer, White, Male). Further, staff also identified the interplay between physical and mental health as indications of successful treatment endorsed by patients.

I was speaking to a couple [of] patients earlier and one of them had said that he or she was able to recently stop taking their blood pressure medication, because he or she has reached a point of peace of mind or a certain level of mental health that they hadn’t had before. They realized that it was through their commitment to mindfulness and those centering practices not to let the stress of daily life overwhelm them. That was really key.

(Interview, Staff, White, Female).

Notably, staff described that patients’ success in treatment included a renewed sense of value and self-worth, particularly for individuals who have experienced past traumas, racism, and stigma.

Primarily, the theme that seems most predominant is when they describe value in their lives and no longer wanting to take risks with their lives, […] understanding that they matter… There are just so many layers of stigma that can be attached to basically being a poor person of color in Baltimore City with a heroin addiction. And if you add trauma, suffering any kind of violence or exposure to violence on top of that, it’s a lot for any one person to handle.

(Interview, Staff, White, Female).

### 3.2. Theme 2: productivity and accomplishment

Both participant groups described various subthemes relating to productivity and accomplishments as indications of treatment success. Emerging subthemes included financial goals, a sense of accomplishment, feeling busy, employment, improvements in legal outcomes, feeling back on track, increased self-value, decreased risk-taking, and autonomous decision-making. While both groups were aligned on many subthemes, there were divergences in some notable areas.

#### 3.2.1. Patient perspectives

Succeeding in methadone treatment included a sense of increased productivity for patient participants. Gaining and maintaining employment, working towards financial goals, and feeling busy and accomplished were cited as examples of successes. In terms of employment and productivity, one participant described doing well in treatment as “[getting] my life back on track, becoming a productive member of society again, [and] working.” Patients also described success as “doing something with my time…keeping myself busy.” Of note, only patient participants endorsed feeling busy as a product of treatment success.

Employment gains, including stability across several life domains, emerged as another success metric for patient participants. Financial and housing stability also stood out to participants as significant successes, as indicated by a patient who stated: “One of the best things is I have more money to do things, productive things, and also, I just started a new job” (Interview, Patient, White, Male). When asked what was important in treatment, this patient endorsed “keeping my job” and highlighted the importance of “[keeping] my place to stay.” Other patients described success as “saving money instead of spending it” and “holding money a lot better.” Patient participants also discussed success as restoring their credit rating, paying down debts, and establishing newly attainable financial goals like buying a house. One patient commented, “Instead of having to use the money for drugs, [I] use the money for other things like clothes and food. It’s doing positive things” (Interview, Patient, White, Male).

Patients identified treatment success as “getting back on track,” which included a return to prior values and goals established before active substance use, and a return to a previous lifestyle perceived as less challenging and more desirable. A patient described this as “getting my life back together and getting back on the right track because I was clean for 11 years, so I know how it feels to live on the other side” (Interview, Patient, Black/African American, Female). Another patient observed:

The rewards you get as you go along in recovery. The positive benefits that come with recovery in sobriety. All the positive things that are better are going to come with it if you do what you’re supposed to do. Life will be easier. The rewards of life being the way it’s supposed to and just not hectic chaos.

(Interview, Patient, White, Male).

Along with productivity and financial rewards, patients cited a sense of accomplishment and progress in their recovery journey as success. A patient indicated, “It’s more than just the act of not using. It’s a feeling of finally completing something, accomplishing something good instead of something bad” (Interview, Patient, Black/African American, Female). Another patient shared, “It feels like I was in school, and I got a good grade on a test like a[n] A plus” (FG, Patient, Native American, Male). Patients perceived a sense of accomplishment derived from commitment and perseverance as gains in treatment.

#### 3.2.2. Staff perspectives

Like patients, staff participants observed patients describing success as getting back on track, including elements like returning to old values, being more active in one’s communities, achieving housing stability, and experiencing increased self-worth and accomplishment.

They reach some point of gaining some stability, rather than being out there and constantly using and not having any place to go, whether or not they’re in some kind of transitional recovery home or back with their family, you know, they’ve reached a level of stability.

(FG, Staff, Black/African American, Female).

Staff also described patients’ treatment success in terms of employment and meeting financial goals. However, only staff described the resulting sense of self-value and decreased risk-taking as consequences of treatment success. Staff noted, “For some clients, their success is a job, a house, some type of income. That’s usually what they think is gonna be successful. The more they start to realize their self-worth, the better they start to feel about themselves” (FG, Staff, Black/African American, Male). Another staff member stated:

Primarily, the theme that seems most predominant is when they describe value in their lives and no longer wanting to take risks with their lives. […] Those who consistently continue to say that now I understand that my life has value, I understand that I matter and that they are doing this for themselves.

(FG, Staff, White, Female).

### 3.3. Theme 3: social improvements

Participants described social improvement as a critical factor resulting from treatment success. Subthemes relating to social improvement included helping others in treatment, environmental changes, recognition for accomplishments, strengthening family relationships, withstanding social substance use influences, increased community engagement, and building or amending healthy relationships. Patient and staff groups were aligned on approximately half of these subthemes, with each group endorsing aspects of social improvements not discussed by the other.

#### 3.3.1. Patient perspectives

Improvements in personal relationships and social dynamics arose as integral components of treatment success for many patients. These included strengthened relationships with family, improved intimate relationships, positive changes to one’s environment, helping others, and being around others who use substances without partaking themselves.

Patients discussed successful treatment as engaging in quality time with family and being a stable figure for their families, exemplified by “being there for my kids, my grandkids, and my great-grandkids” and “calling home every day.” Family improvements also included being accepted and welcomed by family, a stark contrast from the deterioration of close relationships when, as described by one participant, “nobody wants [s] you to be around if you [are] doing drugs.” Patient participants discussed improvements in intimate relationships, such as creating “a better relationship with my girlfriend because she’s a lot happier now since I started on [methadone treatment].” Improvements in social relationships also included positive changes in how patients interact with others by communicating more effectively.

I can see the things that I missed and apply them to my life. [Treatment] helped me to change for the better. Because I was the person that always had something [to] say. I just had to have the last word. But since I’ve been back [in treatment]… it’s helped me to deal with people in society, differently and better.

(Interview, Patient, Black/African American, Female).

Patient participants differed from staff participants in their endorsement of helping others and withstanding social substance use influences. For some, reaching a point in their treatment where they could inspire and “help others do the same” embodied treatment success. Additionally, modifying one’s social circles to “avoid those getting high” further signaled treatment success for many.

#### 3.3.2. Staff perspectives

Staff endorsed many of the same subthemes as patients, including building healthy relationships, renewing family ties, and changing one’s social environment. When describing environmental changes, staff described success as “not associating with people who were their downfall in the past”. Staff echoed patients’ endorsement of renewed relationships as strong indications of strides in treatment.

It’s all about reconnecting and rebuilding relationships, and generally what I’ve heard of a person that’s successful in recovery regardless of methadone or not, they’re getting reconnected back to work, they’re getting reconnected back to their family, back to intimate relationships. That’s when I hear the success.

(FG, Peer, White, Male).

Staff participants endorsed some subthemes not addressed by patients. Alongside improving one’s environment, only staff discussed patients “re-engaging in the community.” Staff also noted success as social recognition and receiving praise from peers due to treatment gains and accomplishments. Staff participants stated that patients exhibited pride in earning take-home bottles and described the importance of showing others that “they’ve done something and achieved something.”

### 3.4. Theme 4: substance use changes

Patient and staff participants agreed on almost all themes relating to substance use changes, with the notable distinction of staff endorsing success as a return to recovery after a period of increased substance use, as well as the experience of long-term reductions in substance use and declining substance use consequences.

#### 3.4.1. Patient perspectives

Patient participants described reductions in or cessation of substance use, including decreased problems associated with substance use and changes in behaviors related to substance use, as part of treatment success. Specifically, persistence when reducing substance use arose as an important outcome to patients. Though abstinence was not the goal for all, it was for many; for instance, one patient outlined how “doing well” meant being “consistent, staying away from drugs.” Another participant stated that treatment success meant they “still haven’t been using.” Patients also described success as the ability to help others working towards the goal of substance use reduction or abstinence. The rewards of recovery included “sobriety and keeping clean and helping others do the same.”

Patients explained how a reduction in urges to use substances equated to treatment success, stating treatment succeeded when it “took away cravings.” With the decrease in cravings came the relief of no longer “being stressful, worrying about getting up, going out there, worrying about getting money, trying to get your blast” to ward off withdrawal symptoms. One patient said, “I just don’t have to worry about looking for drugs every morning” (FG, Patient, White, Male). Clinical indicators of reductions in substance use, including substance-free urinalysis test results, further signaled treatment success to patients. As one patient reflected, “When you get your results from taking a urine test or something and it’s good, you feel good” (FG, Patient, Native American, Male).

#### 3.4.2. Staff perspectives

Staff participants observed many of the same subthemes of substance use changes as patients, including a reduction in desire or cravings for opioid and non-opioid substances and cessation of substance use, which also contributed to patients’ pride in their recovery process.

For a lot of my clients, they’re more proud of themselves because they lasted that long. If you can just make it a week or two without even thinking about going out there and using and really acting on it— we all think about it sometimes, but just acting on it— I think it makes a difference. And I think that’s the most successful part of methadone for them because they don’t really have that urge anymore to go out there and just chase drugs all day. And I think that’s the success part of it. I really do. I see it every day.

(FG, Peer, Black/African American, Female).

Staff also discussed patients’ perception of a methadone treatment program as a pathway to reducing non-opioid substance use.

I know for some of them it’s if they cut back on one [substance] because even though it’s some that are not using it’s a lot that are using everything including opiates… when we talk about cutting back or stopping, even if they stop the marijuana or even if they at least stop the fentanyl, that’s success if we’re talking harm reduction… that’s how they look at it… they don’t want everything taken away like all at once and they just see sometimes gradual steps and that’s fine.

(FG, Staff, Black/African American, Female).

Only staff participants endorsed success as identifying recovery progress, returning to recovery after re-engaging in prior levels of substance use, as well experiencing long-term reductions in substance use and the associated consequences. One staff member indicated a person in recovery is “gonna have some setbacks and being able to see that they have reached some level of improvement, whether they used 10 weeks ago or yesterday and got back on track… That’s success” (FG, Staff, Black/African American, Female).

### 3.5. Theme 5: treatment engagement

All participants agreed that behaviors relating to treatment engagement indicated treatment success. Though both groups were largely in agreement on this theme, only providers described future-oriented thinking and long-term treatment planning as part of patients’ experiences in successful treatment.

#### 3.5.1. Patient perspectives

Patients identified an outcome of successful methadone treatment as the act of commitment and consistency in one’s recovery. A high level of engagement with and commitment to methadone treatment included having goals in treatment and consistency in one’s dosing schedule. Patients endorsed success when they remained in the methadone treatment program, particularly after experiencing past treatment attempts. As elaborated by a patient, “This is the third time I’ve been on the methadone program. The first two I walked off, because I really wasn’t ready. But this time I think I’m actually doing wonderful because I stayed on for over a year” (Interview, Patient, Black/African American, Female).

Some participants discussed successful methadone treatment as fully engaging in treatment to the point of no longer necessitating MOUD. For example, when asked what doing well in methadone treatment looked like, participants endorsed “walking away from it,” “a slow detox and be out of here,” and “hopefully, by next year, I won’t even be on the program anymore.”

#### 3.5.2. Staff perspectives

Staff also observed patients describing successful methadone treatment as increased levels of engagement in one’s treatment and recovery journey. Engaging with methadone treatment as a form of treatment success meant not only following a prescribed dosing plan but also included involvement in comprehensive engagement in care, such as meeting with staff and becoming involved in programs available at the clinic.

Success means that [the patient is] coming to treatment every day to get dosed, and getting dosed and participating in treatment as recommended… But just getting here and participating in not only your dosing, meeting with your counselor, meeting with the doctors, following through with the study programs that are here, just engaging in treatment altogether.

(FG, Staff, Black/African American, Female).

Rewards within methadone treatment stood out to staff as success, including reaching treatment milestones such as “getting a take-home bottle because that means that they’ve done something to earn it*”* (FG, Staff, Black/African American, Female). Staff also defined success as one’s ability to maintain their recovery if they disengaged from methadone treatment.

When they start weaning off, start coming down to the realization that they can do what they need to do for themselves, for their family, without it. I’ve seen the process of individuals who weaned off of methadone and they got back to being [those] individuals without it.

(FG, Peer, Black/African American, Male).

Further, success included learning from past experiences in methadone treatment, returning to treatment after prior attempts, and the ability to recognize which components of treatment work best in each individual’s unique recovery pathway.

Part of the success story looks like to me is to see that a person may have tried one area of treatment, and it didn’t work… Maybe you did see something else that worked better for you and once you saw that, once you realized that, and once you continued with that and you stuck to it, that’s part of a success story to me.

(FG, Staff, Black/African American, Female).

According to staff, being proactive about treatment goals, maintaining dosing stability, regular treatment attendance, commitment to treatment, as well as future-oriented thinking factored into patients’ perceptions of success. Though staff agreed with patients on almost all subthemes, only staff discussed future-oriented thinking as an indication of success.

Success looks like a member who is stable in their doses, re-engaging in the community, not mixing or interacting [with] other drugs along with their medication, and has created a plan for their self on whether how long they’re going to be on the methadone. They’re looking for the future, where they’re going with this treatment.

(FG, Peer, Black/African American, Male).

## 4. Discussion

This study aimed to capture the voices of patients and staff (staff and PRSs) who defined treatment success at a Baltimore-based methadone treatment program. We examined how patients and staff described successful treatment outcomes and explored the levels of congruence between patients’ conceptualization of success and staff perspectives. We applied the findings of this study utilizing the HEIF to propose opportunities to implement person-centered definitions of successful methadone treatment to improve health equity and treatment outcomes. To our knowledge, this is the first study to highlight the perspectives of patients of predominantly low-income, racially minoritized backgrounds and staff of a methadone treatment program, contributing to more representative definitions of person-centered MOUD treatment success.

Patient and staff participants’ conceptualizations of successful methadone treatment outcomes were generally aligned with some distinctions (see Table 2). Both patients and staff described success in methadone treatment across five overarching areas: improved general health, productivity and a sense of accomplishment, social improvements, substance use changes, and treatment engagement. These findings align with previous reports of patient and staff endorsement of success (Hooker et al., 2022; Shadowen et al., 2022).

Unlike previous reports, however, our findings revealed distinct differences in the subthemes identified by patients and staff. When describing accomplishments as a form of treatment success, patients described feeling busy and engaging in more activities. In contrast, only staff highlighted an increased sense of self-value and reduced risk-taking behaviors. Patients also identified helping others in treatment and resisting social substance use pressures as crucial markers of success. Meanwhile, staff emphasized receiving recognition for treatment progress and increasing community engagement. When discussing substance use, staff exclusively mentioned long-term reductions in substance use, along with fewer substance-related consequences, as well as the importance of returning to recovery after a period of increased substance use. Regarding treatment engagement, only staff noted long-term recovery planning and future-oriented thinking as indicators of success. The subthemes addressed by the patient group presented a stronger focus on more immediate strategies, emphasizing activity levels, personal autonomy, positive social influence, resilience, and self-agency in daily activities. In contrast, the subthemes only discussed by staff reflected a focus on long-term recovery aims, with sustained behavioral change, internal aspects of success like self-value, and external markers of recovery such as social recognition. These differences highlight areas of contrast between patient and staff perspectives which may have implications for revisiting patient-staff alignment around the foundational motivations and goals driving engagement in care.

While many distinctions were apparent in patient and staff perspectives, the converging definitions identified by staff in this study may be attributable to the harm-reduction approach and policies adopted by the Baltimore-based study site clinic, where the first-line approach to supporting patients with continued substance use is to reevaluate a treatment plan. In contrast, many treatment programs initiate treatment termination and administrative discharge. Nonetheless, informative discrepancies in describing successful treatment arose between patient and staff participants, presenting the opportunity to fine-tune person-centered care to be most responsive to patient goals.

Through the lens of the HEIF, the findings of this study point to a need to reexamine success in methadone treatment as defined by patients, particularly incorporating the underrepresented voices of low-income, marginalized individuals in care. A focus must be placed on the person-centered motivations driving patients to seek out and engage in care, and this focus must be leveraged to tailor treatment to the individual and optimize the clinical experience. For example, individual patient factors such as personal goals, purpose, and circumstances should be considered by staff at the outset of treatment planning and throughout treatment monitoring. The characteristics of methadone treatment itself, such as the demands and scheduling of treatment, should be evaluated to maximize the feasibility of patient success. The clinical encounter must be a collaborative effort where patients are empowered and engaged in treatment planning, and staff are responsive to patient-defined goals. Clinic, hospital, and healthcare policies must broaden traditional conceptualizations of success, examine patient experiences of meaningful treatment outcomes, address staff stigmas and misconceptions, and operationalize person-centered methadone treatment in practice. Importantly, local, state and federal policy must be responsive to the needs of patients in methadone treatment, where stringent and exclusionary approaches to treatment can alienate patients, limit treatment access and patient engagement, and threaten adverse health outcomes.

**Fig. 1.**
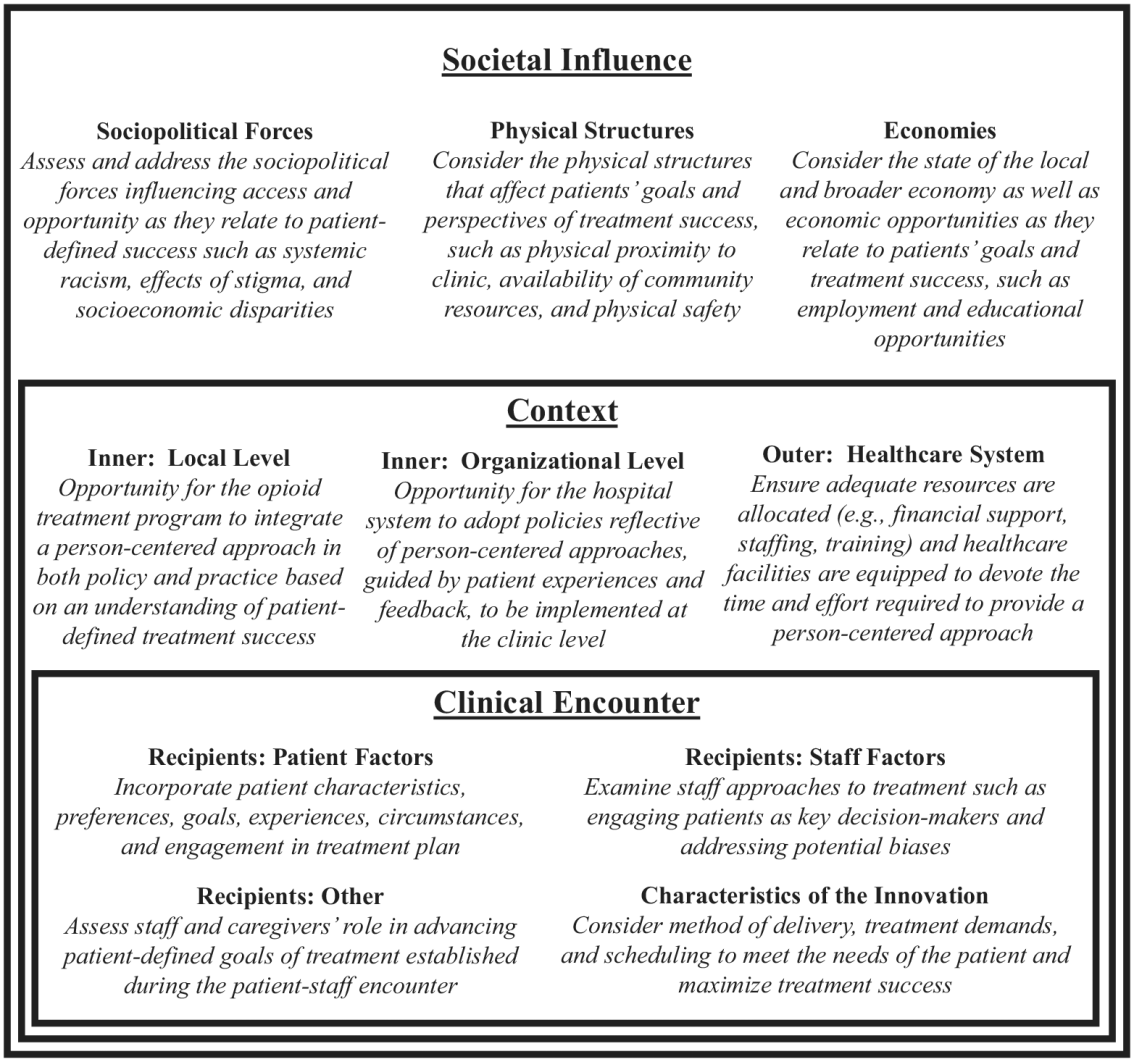
Adapted HEIF Model* – Opportunities to Incorporate Person-Centered MT Success. *Figure adapted from “The health equity implementation framework: proposal and preliminary study of hepatitis C virus treatment,” by E. Woodward, M. Mattieu, U. Uchendu, et al., 2019, *Implementation Science,* 14, 26. doi:https://doi.org/10.1186/s13012-019-0861-y.

While an in-depth analysis of implementing person-centered definitions of methadone treatment success is beyond the scope of this work, we propose opportunities to integrate person-centered definitions of success in research, policy, and practice, per the HEIF, to offer more responsive, representative, and equitable treatment for patients engaged in MOUD care. The recommendations in Fig. 1 highlight areas to integrate person-centered treatment goals within the broader individual, organizational, and societal contexts of the HEIF. These recommendations reflect the qualitative findings of this work within the established principles of the HEIF model, setting the stage to incorporate patient perspectives and experiences to inform more equitable and representative treatment implementation.

Person-centered definitions of successful treatment outcomes expressed by participants of this study may help inform future research seeking a more representative measurement of methadone treatment success. Incorporating the perspectives of low-income, marginalized patients in methadone treatment can offer a more representative and responsive measure of MOUD success. Such changes could result in enhanced treatment experiences, improved treatment engagements, and more equitable outcomes for patients in MOUD care (Baumann & Cabassa, 2020; Sanger et al., 2022). Understanding patient perspectives in MOUD has become a paramount issue at the federal level, with newly established SAMHSA regulations setting the stage for person-centered MOUD care. Future studies should explore person-centered definitions of success across MOUD treatment types and propose a more representative measurement of treatment success to inform research, practice, and policy implementation. We hope the findings of this study present opportunities to continue such an effort.

### 4.1. Limitations

This study included methodological limitations that should be addressed in future research. Though we employed procedures to reach patients both consistently and inconsistently attending methadone dosing visits, enrollment at a treatment site meant that patient participants were all actively receiving treatment in the methadone treatment program. Therefore, we were unable to capture the perspectives of patients who discontinued and have not returned to methadone treatment. We also recognize the limitations of recruiting patients and staff from one treatment program and one MOUD modality (methadone treatment), as well as the lack of patients who self-identified as Asian, Hispanic, Pacific Islander, or other racial identities not represented within this sample.

We also included different data collection methods (individual interviews and focus groups) based on stakeholder input to promote comfort and participation. However, we cannot guarantee that participants did not withhold information about their experiences and opinions in either the focus group or interview setting considering the sensitive nature of the topics covered. Also, small sample sizes within each group (i.e., patients, staff) did not allow for formal comparisons across groups and within the staff group (PRSs versus different staff roles), which may have limited the scope of responses captured in this study. While we could identify concordance between groups, the sample size of this study limits our ability to conclude the extent to which thematic discordance reflects patient-staff disagreement. We believe this work provides an important starting point for examining the themes and subthemes endorsed across patients and staff, and which were omitted or emphasized in one group but not the other.

While our team determined the focus groups and interviews were sufficient to capture the five key themes emerging from participant feedback across groups with no indication of additional themes emerging, we cannot be sure that new subthemes would not have presented with further data collection. As this work enabled us to discern the presence or omission of subthemes between groups and to draw broad categories of themes addressed by each group, we believe this work provides an important starting point for examining the types of themes endorsed by both groups and the endorsement or omission of themes between groups. Future research would benefit from recruiting larger sample sizes to acquire additional data and conduct a more rigorous comparison.

Finally, though we asked staff to describe successful treatment outcomes based on patient feedback, staff responses may be biased by their perspectives. For some staff who endorsed prior substance use, their responses may also be influenced by past lived experiences with substance use and treatment. While these influences limit our ability to distinguish between solely patient or staff perspectives, we believe these perspectives still contribute to a person-centered evaluation of treatment success.

Despite these limitations, we feel confident in the validity of our findings based on the indication of theoretical saturation and our ability to capture and integrate perspectives of individuals often neglected in research participation (i.e., underserved, low-income, racially minoritized patients struggling with methadone treatment retention and peer staff whose perspectives are not frequently included as part of the care team). Investigations such as the present study, which focuses on this population as the predominant recipients of methadone treatment, offer opportunities for the methadone treatment research, staff, and policy communities to revisit how methadone treatment success is conceptualized, measured, and achieved. A more attuned understanding of the perspectives and experiences of the methadone treatment patient and staff population on the front lines of the opioid crisis could enhance the treatment experience, improve retention, and reinforce success.

Future studies should integrate quantitative assessments of the various conceptualizations of success presented in this study with larger sample sizes. Further, future works should identify if and how the conceptualizations of treatment success presented in this study change over time spent in MOUD treatment. Researchers should also examine the distinctions in success definitions across staff roles (physicians, PRSs, counselors) and how these factors may converge or diverge with patient perspectives of treatment success. Despite these limitations, this study represents a first step in integrating patient and staff perspectives to enhance person-centered care in methadone treatment.

### 4.2. Conclusion

The findings of this study reinforce and expand upon the existing literature on the topic of MOUD treatment outcomes outside of the traditionally held success markers of retention and abstinence, particularly for underrepresented and marginalized individuals engaged in methadone treatment. The patient and staff voices presented here suggest various domains exist when observing successful treatment, including MOUD. The broader clinical and public health implications of these and similar findings include an integrated and more representative conceptualization of person-centered success that can best serve patient interests in treatment. Research, practice, and policy must all account for patient-defined methadone experiences and goals at the individual, clinical, organizational, environmental, and sociopolitical levels. With more tailored treatment planning based on representative definitions of success, patient outcomes and treatment experiences have the potential to improve.

## Figures and Tables

**Table 1:** Sociodemographic characteristics of participants.

	Patients (*n* = 20)	Staff (*n* = 12)	Total sample (*n* = 32)
	M (SD)	M (SD)	M (SD)
**Age**	48.4 (10.0)	49.2 (0.7)	48.7 (10.1)
	n (%)	n (%)	n (%)
**Race**			
Black / African American	12 (60)	9 (75)	21 (66)
White	5 (25)	2 (17)	7 (22)
American Indian / Alaska Native	1 (5)	0 (0)	1 (3)
Other	2 (10)	1 (8)	3 (9)
**Gender** *			
Male	14 (70)	5 (42)	19 (59)
Female	6 (30)	7 (38)	13 (41)
**Education**			
Less than high school	7 (35)	0 (0)	7 (22)
Completed high school	8 (40)	3 (25)	12 (38)
Some college	3 (15)	1 (8)	4 (13)
Associate’s degree	2 (10)	3 (25)	5 (16)
Bachelor’s degree	0 (0)	2 (17)	2 (6)
Master’ s degree	0 (0)	3 (25)	3 (9)
**Substance use and treatment history**			
>1 substance use treatment enrollment	11 (55)	^†^–	–
> 1 MOUD program enrollment	9 (45)	–	
Any history of substance use and recovery	–	10 (83)	–
	M (SD)	M (SD)	M (SD)
Age of first substance use	17.7 (5.1)	–	–
Age of first opioid use	19.3 (5.2)	–	–
Number of prior treatment enrollments	2.6 (3.1)	–	–
Number of prior MOUD enrollments	1.7 (1.6)	–	–
Years in substance use recovery	–	15.6 (8.1)	–
Years working in substance use treatment	–	9.6 (7.6)	–

SD = Standard deviation.

* Of multiple gender options presented on the survey, all participants self-identified as male or female.

† – denotes items not asked.

**Table 2:** Interview and focus group themes and subthemes.

Theme	Sub-theme	Patients	Staff
**General health**	Mental wellbeing	x	x
	Identifying/dealing with health problems	x	x
	Physical wellbeing	x	x
**Productivity and accomplishment**	Financial goals	x	x
	Sense of accomplishment	x	x
	Feeling busy	x	
	Employment	x	x
	Stable housing	x	x
	Legal outcomes	x	x
	Back on track	x	x
	Sense of self-value		x
	Decreased risk-taking		x
	Autonomous decision-making	x	x
**Social improvements**	Positive influence and helping others	x	
	Changing environment	x	x
	Receiving recognition for accomplishments		x
	Family relationships	x	x
	Resisting social substance use influences	x	
	Community engagement		x
	Building/amending healthy relationships	x	x
**Substance use changes**	Abstinence	x	x
	Return to recovery		x
	Long-term reduction of substance use and consequences		x
	Reduction in want or need of drugs	x	x
	Reduction in non-opioid substance use	x	x
	Reduction in cravings	x	x
**Treatment engagement**	Engagement with psychosocial services	x	x
	Treatment planning	x	x
	Adherence to dosing schedule	x	x
	Future-oriented thinking		x
	Maintaining stable dose	x	x
